# Supplementary material for: Healthcare providers experiences with shared medical appointments for heart failure
Source: PLoS One. 2022 Feb 7;17(2):e0263498. doi: 10.1371/journal.pone.0263498 (PMC8820643; doi:10.1371/journal.pone.0263498)
Supplement: S1 Appendix — (DOCX) [file pone.0263498.s001.docx]

## S1 Appendix A. Interview Guide

*Hello, my name is XX and I would like to thank you for agreeing to participate in our study. I will be asking you some questions and [note taker name] will be taking notes today. We will be audio recording this interview, if that ok with you? If ok, I will ask the question again so that we have a recording of your agreement on the file. (Turn on the audio recorder and re-ask the question)*

*I would like to ask you a few questions about your perceptions of how the shared medical appointments, sometimes referred to as group visits, for heart failure may help you care for your patients who have been hospitalized for chronic heart failure.*

*Your participation is voluntary. You may stop the interview at any time. Your responses will be kept confidential, all responses will be compiled as a group and used in research reports and publications; you will not identified.*

1. First could you tell me what you know about shared medical appointments for heart failure intervention?
2. Have any of your patients participated in the SMA for heart failure? (if no, skip to Question 5)
3. In what ways, if at all, do you find the SMAs for heart failure to be helpful to your patients? (If response is that they are not helpful, ask : Why aren’t they helpful to your patients? Then: can you think how to improve the SMAs to be more helpful to your patients?)
4. How are the SMAs for heart failure helpful to you, if at all, in taking care of your patients with heart failure? (If response is that they are not helpful, ask : Why aren’t they helpful to you? Then: can you think how the SMAs could be improved to be more helpful to you?)
5. What is your overall opinion about shared medical appointments for heart failure?
6. Do you refer patients to the SMA’s? If yes, what types of patients do you refer to this program?

Before we end I want to give our note taker the opportunity to ask any clarifying questions.

***Thank you very much; we know you’re very busy and we appreciate the time you’re taking to help with this study.***
