# Supplementary material for: Healthcare providers experiences with shared medical appointments for heart failure
Source: PLoS One. 2022 Feb 7;17(2):e0263498. doi: 10.1371/journal.pone.0263498 (PMC8820643; doi:10.1371/journal.pone.0263498)
Supplement: S2 Appendix — (DOCX) [file pone.0263498.s002.docx]

# Agenda for Debriefing Post Interview Meetings

**Interview summary**

**Recruitment**

**Interview**

**Immediately following interview whenever possible.**

| 1. **In general HOW DID THE INTERVIEW GO? (e.g. how engaged was participant, how candid were responses, how was your own comfort level, were there interruptions):** |
| --- |
| 1. **What were the MAIN THINGS that came up? (brief points):** |
| 1. **Did anything NEW come up?** |
| 1. **Did anything SURPRISE you?** |
| 1. **Did the interviewee show signs of interview fatigue?** |
| 1. **Additional Considerations?** |
| 1. **Was anything CONFIRMED or CHALLENGED for you after this interview?**   Challenged:  Confirmed: |
| 1. **Should we CHANGE ANYTHING** |
| 1. **PI /Team comments** |
| 1. **Any follow up with medical team. What type of information from interviews is or is not appropriate for us to communicate back to medical and/or entire study team?** |
