# Supplementary material for: Healthcare providers experiences with shared medical appointments for heart failure
Source: PLoS One. 2022 Feb 7;17(2):e0263498. doi: 10.1371/journal.pone.0263498 (PMC8820643; doi:10.1371/journal.pone.0263498)
Supplement: Attachment — Submitted filename: PLOS ONE R2.docx [file pone.0263498.s004.docx]

Dear Editor-in-chief,

Thanks for allowing me to evaluate the revised version of the paper entitled "Healthcare providers experiences with shared medical appointments for heart failure". I believe the authors did a good job addressing all points addressed from my side.

- The introduction was further expanded to include relevant suggested information.
- The methods section was greatly improved to make it possible to duplicate the study in the future.
- Conclusions are presented in appropriate way and are congruent with the study findings. The authors reconstructed this section based on the results as suggested by the reviewer.
- The authors copyedited the paper to improve the flow of information.

I do believe that the article is now presented in intelligible fashion that meets standards of rigor scientific research.

Kind regards,

Yours sincerely,

Rawand A. Khasawneh

Assistant Professor of Clinical Pharmacy

Faculty of Pharmacy

Jordan University of Science and Technology, Irbid-Jordan
